# 

**Published:** 1895-07

**Authors:** 


### CONTENTS—JULY, 1895.—VOL. XI., NO. 1.

ORIGINAL COMMUNICATIONS—

Fæcal Fistulæ and Artificial Anus. Prof. J. E. Thompson, M. D., Galveston . . . . . 1
The Menopause, its Complications and Treatment. N. A. Olive, M. D., Waco . . . . . 9
Clinical Investigations with Regard to the Therapeutic Properties of Tannigen. Dr. Karl Kunkler . . . . . 17
Ununited Practures. J. R. Stuart, M. D., Houston . . . . . 27
A Case of Hysterectomy for the Removal of an Enormously Large Fibroid Uterus. J. H. Evans, M. D., Palestine . . . . . 32
Report of Thirteen Operations for Goitre, and Technique employed. Dr. B. E. Hadra, San Antonio . . . . . 33

ABSTRACTS AND SELECTIONS—

The Etiology and Treatment of Inflammation of the Uterine Appendages . . . . . 35
"Hold Out Your Tongue" . . . . . 36
The Sossige . . . . . 36

EDITORIAL—

Regulating the Practice . . . . . 37
"Miasmatic Paralytic Fever" . . . . . 38
Dr. Peeples' Article . . . . . 40

MEDICAL NEWS AND MISCELLANY—

Numerous Paragraphs of Interest . . . . . 41
A Naraow Escape . . . . . 41

BOOK NOTICES— . . . . . 42

PUBLISHERS' NOTES . . . . . 45

